# Discovery of a novel Aurora B inhibitor GSK650394 with potent anticancer and anti-*aspergillus fumigatus* dual efficacies *in vitro*

**DOI:** 10.1080/14756366.2021.1975693

**Published:** 2021-12-11

**Authors:** Yuhua He, Wei Fu, Liyang Du, Huiqiao Yao, Zhengkang Hua, Jinyu Li, Zhonghui Lin

**Affiliations:** College of Chemistry, Fuzhou University, Fuzhou, China

**Keywords:** Aurora B, inhibitor, anti-cancer, anti-aspergillus fumigatus

## Abstract

Invasive fungal infections including Candidiasis and Aspergillosis are associated with considerable morbidity and mortality in immunocompromised individuals, such as cancer patients. Aurora B is a key mitotic kinase required for the cell division of eukaryotes from fungus to man. Here, we identified a novel Aurora B inhibitor GSK650394 that can inhibit the recombinant Aurora B from human and *Aspergillus fumigatus*, with IC_50_ values of 5.68 and 1.29 µM, respectively. In HeLa and HepG2 cells, GSK650394 diminishes the endogenous Aurora B activity and causes cell cycle arrest in G2/M phase. Further cell-based assays demonstrate that GSK650394 efficiently suppresses the proliferation of both cancer cells and *Aspergillus fumigatus*. Finally, the molecular docking calculation and site-directed mutagenesis analyses reveal the molecular mechanism of Aurora B inhibition by GSK650394. Our work is expected to provide new insight into the combinational therapy of cancer and *Aspergillus fumigatus* infection.

## 1. Introduction

Cancer patients undergoing radiotherapy or chemotherapy are often severely immunocompromised. These patients are particularly vulnerable to invasive fungal infections, which are associated with considerable morbidity and mortality^1^. As suggested by the National Comprehensive Cancer Network (NCCN) clinical practice guidelines, primary antifungal prophylaxis should be used in high-risk patients. Candidiasis and aspergillosis are among the most common fungal infections in immunocompromised hosts^1^^,^^2^. Recently, the increasing resistance to current antifungal drugs such as Azoles has caused serious clinical problems ^3^. Therefore, there is an urgent need to develop new antifungal drug targets.

Mitosis is a fundamental biological process essential for the growth and development of all eukaryotes, including fungus and humans. The serine/threonine kinase Aurora B is a pivotal mitotic regulator conserved from yeast to man and involves various aspects of cell division, such as chromosome condensation, kinetochore functions, spindle checkpoint activation, and cytokinesis completion^4^. Aurora B physically associates with the inner centromere protein (INCENP), borealin and survivin to form the chromosomal passenger complex (CPC)^5^^,^^6^. As a kinase, it can phosphorylate many proteins, including histone H3^7^, mitotic centromere-associated kinesin (MCAK)^8^, and centromere protein-A (CENP-A)^9^. The overexpression of Aurora B is frequently observed in many cancers, including myeloma^10^, colorectal^11^, prostate^12^, pancreatic^13^, hepatocellular^14^, and ovarian^15^ carcinomas. It has been clinically linked to poor patient prognosis in cancers^16^. In contrast, reducing the expression of Aurora B kinase by short hairpin RNA (shRNA)^17^ or chemical inhibitors^4^^,^^18–24^ has been demonstrated to prevent tumour cells growth. For example, the highly selective Aurora B kinase inhibitor barasertib (AZD1152) exhibited an overall response rate of 45% in phase I/II trials in patients with acute myelogenous leukaemia (AML) when combined with low-dose cytosine arabinoside (LDAC)^23^. The selective pan-Aurora kinase inhibitor AMG 900 displayed potent inhibitory activity against a range of human solid tumour cell lines^24^. The Aurora kinases inhibitor VX680 has been shown to suppress the growth of HepG2 cells^25^. Thus, Aurora B has been a promising drug target for the treatment of many cancers, including AML^23^, osteosarcoma^20^, and myeloma^10^.

Anti-mitosis is exploited not only for anti-cancer treatments but also for anti-fungal therapies. For example, Griseofulvin, a medication used to treat many types of dermatophytoses, has been shown to inhibit the progression of mitosis in fungal cells by suppressing spindle microtubule dynamic^26^. Furthermore, the fact that Aurora B homologue Ipl1 kinase is essential for viability in budding yeast suggests that Aurora B is absolutely required for yeast cell proliferation^27^. Thus, Aurora B could also be a promising target for developing antifungal agents.

The compound GSK650394 was previously synthesised by GlaxoSmithKline and developed as an inhibitor of serum- and glucocorticoid-regulated kinases, SGK1 and SGK2^28^. It has been shown that GSK650394 possesses antiproliferative properties against many cancer cell lines including lung cancer^29^, thyroid cancer^30^, and human prostate cancer^28^. In this study, we performed a high-throughput small-molecule screen of about 8,000 compounds against the ATPase activity of human Aurora B followed by a validation of anti-cancer effects *in vitro*, and a secondary screen for anti-*Aspergillus fumigatus* activity. These screens identify GSK650394 as a novel Aurora B inhibitor with potent anti-cancer activity in HeLa and HepG2 cell lines and anti-proliferation activity in *Aspergillus fumigatus* human pathogenic fungal cells. Our findings are thus expected to provide new insight into the combinational treatment of cancer patients with *Aspergillus fumigatus* infection.

## 2. Materials and methods

### 2.1. Compound, antibodies and cell lines

All compounds were purchased from TopScience (Shanghai China). Anti-histone H3 and anti-histone H3 phospho-Ser10 (pH3) antibodies were purchased from Cell Signalling Technology (USA). HeLa and HepG2 cell lines were obtained from the Cell Bank Shanghai, China.

### 2.2. Protein expression and purification

The pGEX-dual expression plasmid containing human (*Hs.*) Aurora B kinase domain (aa.54–344) and a C-terminal fragment of *Hs.*INCENP (aa.835–904) was obtained from Prof. Hongtao Yu (Department of Life Science, Westlake University). The cDNAs of *Af.*Aurora B kinase domain (aa.1–396, GenBank accession no. EDP54304.1) and the C-terminal fragment of *Af.*INCENP (aa.1200–1297, GenBank accession no. XP_033417438.1) was synthesised in Union Biotech (China). Protein expression and purification were performed as previously described^31^. Briefly, The recombinant proteins of Aurora B and INCENP were coexpressed in BL21 (DE3) pLysS competent cells. For protein purification, cells co-expressing GST-INCENP and Aurora B proteins were resuspended with lysis buffer containing 50 mM Tris-HCl, pH 8.0, 200 mM NaCl, 5% glycerol, and 0.05% Triton X-100. Cells were disrupted by French Pressure (Union Biotech, China) and the supernatants were collected after centrifugation. The Aurora B/*Hs.*INCENP complex proteins were pooled through Glutathione columns (Smart-Lifesciences, China). The GST tag was removed by homemade precision protease and the untagged proteins were further purified through a Superdex 200 10/300 GL column (GE Healthcare), with a running buffer containing 20 mM Tris-HCl, pH 8.0, 150 mM NaCl, and 1 mM DTT.

### 2.3. *In vitro* kinase assay

The *in vitro* kinase assay was performed as previously described^32^. The kinase activity of recombinant Aurora B was assayed by incubating 200 nM purified Aurora B/INCENP protein with 100 µM ATP and 6 µg bulk histones (from calf thymus, Sigma-Aldrich) in kinase buffer containing 50 mM Tris-HCl, pH7.4, 100 mM NaCl, 10 mM MgCl_2_, 1 mM DTT and 0.1% Tween20. After 15-min incubation at 30 °C, the reaction was quenched by SDS-sample buffer, separated on SDS-PAGE, and immunoblotted with anti-histone H3 and anti-histone H3 phospho-Ser10 (pS10) antibodies. The blots were scanned and quantified with the ChemiDoc™ Touch imaging system (Bio-rad). To investigate the compounds’ inhibitory effect, Aurora B was pre-incubated with DMSO or various concentrations of drugs for 15 min at room temperature and the reactions were then initialised by the addition of ATP and histone substrates.

### 2.4. Luciferase-coupled ATP assay

A luciferase-based luminescence assay was utilised to determine the ATPase activity of Aurora B according to the manufacture’s protocols (Promega). Briefly, the Aurora B protein was incubated with 10 µM ATP in the absence or presence of various drugs at 30 °C, in a kinase buffer containing 50 mM Tris-HCl, pH7.4, 100 mM NaCl, 10 mM MgCl_2_, 1 mM DTT, and 0.1% Tween20. At the end of the reaction, the Kinase-Glo reagent (Promega) was added and the ATPase activity was evaluated by luminescence measurement on the microplate reader (SpectraMax-L, Molecular Devices).

### 2.5. Cell culture, treatments and flow cytometry assay

HepG2 and HeLa cells were cultured in DMEM and RPMI-1640, respectively, supplemented with 10% foetal bovine serum in 37 °C and 5% CO_2_. G1/S synchronisation was carried out by treating cells with 2 mM of thymidine for 19 h. The synchronised cells were then released to the normal culture media in the absence or presence of drugs for 18 h. Subsequently, cells were harvested and subjected to immunoblotting or flow cytometric analysis. For flow cytometry assay, cells were trypsinized, washed once with PBS, and then fixed with cold 75% ethanol. Fixed cells were washed with PBS and resuspended in PBS containing the propidium iodide (Sigma) DNA staining solution, for 30 min at 37 °C. Then, samples were analysed using the Cytoflex cytometer (Beckman Coulter, USA) and the data were processed with the Flow Jo software.

### 2.6. MTT assay

MTT assay was conducted for cell proliferation analysis^33^. Cells were cultured in 96-well plates (6 × 10^3^/well) and treated with drugs or left untreated for 24–72 h. After treatment, cells were incubated with MTT solution (0.5 mg/mL) for 4 h at 37 °C. Then, the reactions were terminated by DMSO and the optical densities were measured by absorbance at 570 nm.

### 2.7. Fluorescence imaging

The fluorescence imaging was carried out to observe the nuclear morphology of cancer cells upon drug treatments. HeLa cells were cultured in 96-well plates (5 × 10^3^/well) in the absence or presence of various concentrations of drugs for 48 h, then the nuclei were stained by Hoechst solution (0.01 mg/mL) and the fluorescence imaging was done using the high-content imaging system (PerkinElmer, USA).

### 2.8. Anti-*Aspergillus fumigatus* assay

A plate assay was carried out to assess the growth rate of *Aspergillus fumigatus*. A specified amount of *Aspergillus fumigatus* spores were inoculated on the centre of the plate containing solid glucose minimal medium in the absence or presence of drugs. After further incubation for 30 h at 37 °C, the growth rate was determined by measuring the diameter of *Aspergillus fumigatus* clonies on each plate.

### 2.9. Molecular docking calculation

The molecular docking of GSK650394 onto *Hs.*Aurora B was performed with AutoDock 4.2 package^34^ using the Lamarckian genetic algorithm^35^. The initial structure of Aurora B was obtained from the PDB database (PDB ID: 4AF3^36^, resolution at 2.75 Å). The geometry of the ligand was optimised using GAMESS^37^ with the B3LYP functional^38^ and 6–31 G(*) basis set^39^. The grid map, centred at Aurora B was calculated using 126 × 126 × 126 points with 0.375 Å spacing. Three-hundred docking runs were carried out. The binding energy and cluster analyses were conducted using the default parameters implemented in AutoDockTools 1.5^34^. The best docking result was defined by the model having the lowest binding energy within the dominant cluster.

## 3. Results

### 3.1. Identification of GSK650394 as a novel Aurora B inhibitor

**Figure 1.**
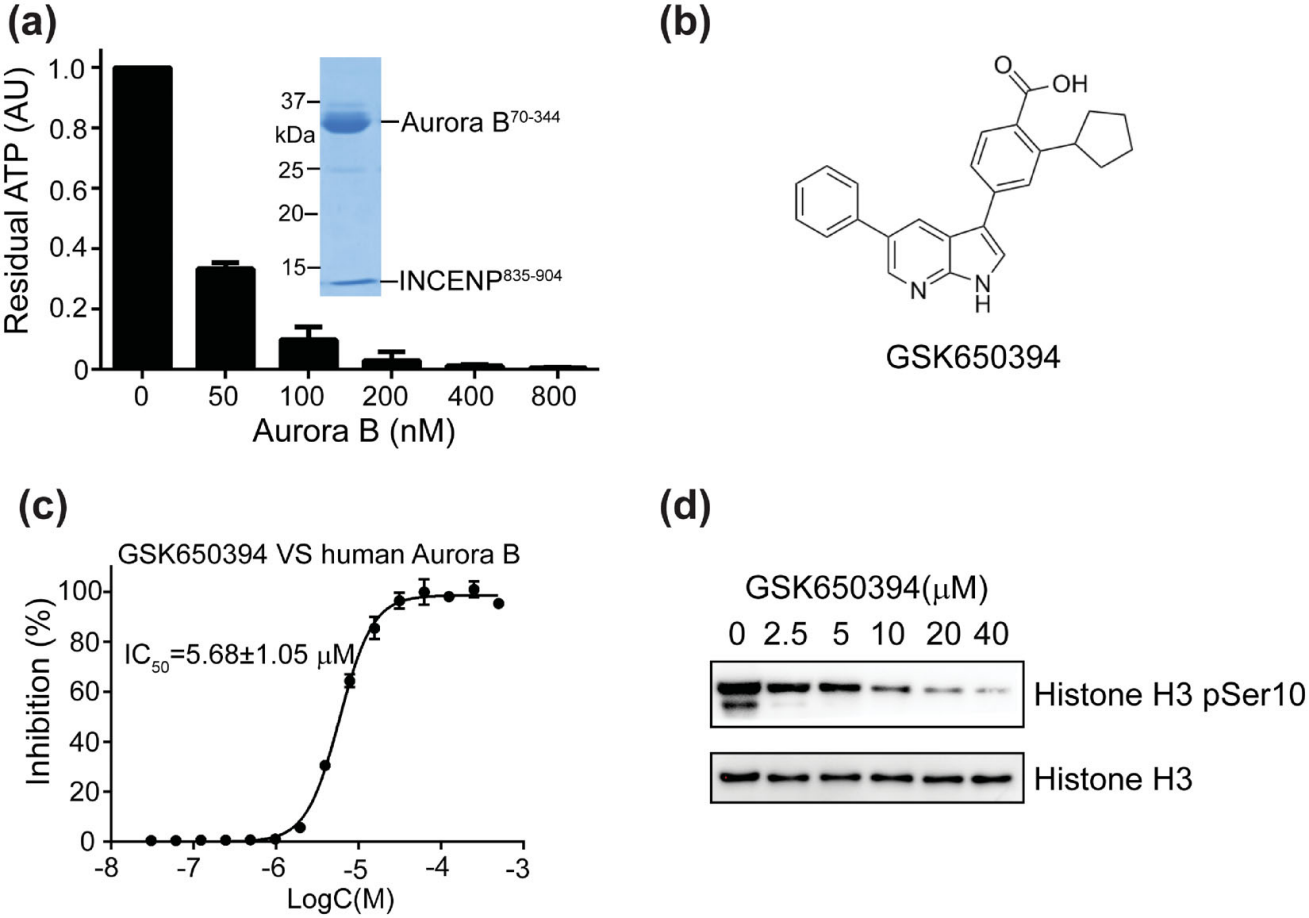
Identification of GSK650394 as a novel Aurora B inhibitor. (a) The ATPase activity of Aurora B was measured by its ability to hydrolyse ATP using the luciferase-coupled ATP assay. The luminescence of the ATP-only control was normalised to 1.0. Values are means ± SD, *n* = 3. SDS-PAGE of the representative eluted fraction of Aurora B/INCENP protein complex is shown in the inset. (b) The chemical structure of GSK650394. (c) Inhibition of the ATPase activity of human Aurora B by GSK650394. The values were normalised to ATP-only control set as 100% inhibition. Values are means ± SD, *n* = 3. (d) Western blot analysis of histone H3 Ser10 phosphorylation (pH3) by human Aurora B in the presence of indicated concentrations of GSK650394. The immunoblots are representative of two independent experiments.

**Figure 2.**
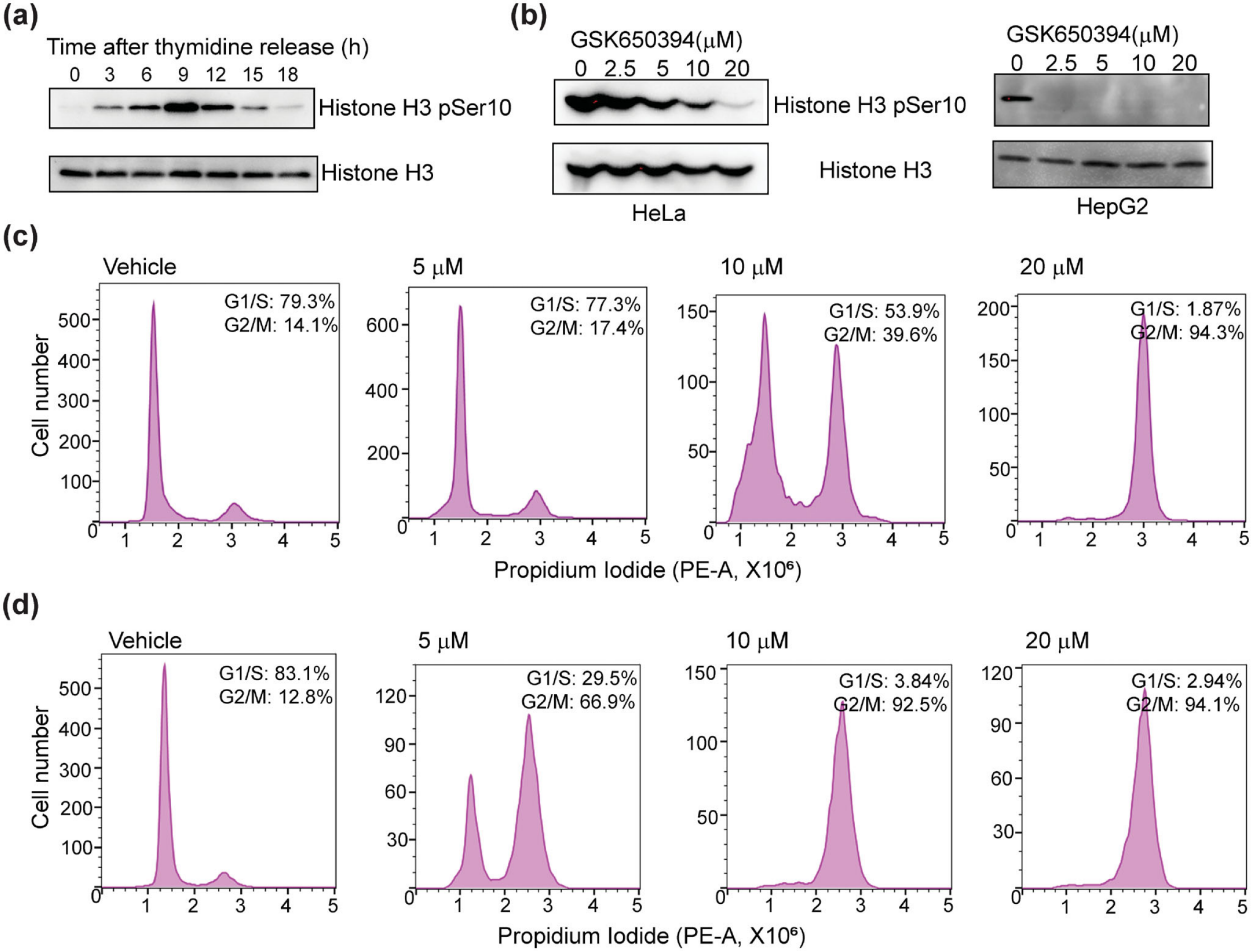
Effect of GSK650394 on cell cycle progression. (a) The dynamic change of histone H3 phosphorylation during the cell cycle. Cells were synchronised with thymidine and released into normal media for the indicated time, followed by immunoblotting with indicated antibodies. The immunoblots are representative of three independent experiments. (b) Effect of GSK650394 on histone H3 phosphorylation in HeLa and HepG2 cells. The thymidine arrested cells were released into media without or with indicated concentrations of drugs for 9 h. Samples were blotted with indicated antibodies. The immunoblots are representative of three independent experiments. (c, d) Effect of GSK650394 on cell cycle progression in HeLa (c) and HepG2 (d) cells. G1/S synchronised cells were released into media without or with indicated concentrations of drugs for 20 h, the resulting cells were subjected to cell cycle analysis by flow cytometry assay.

To obtain active enzyme, the *Hs.*Aurora B kinase domain (aa.70–344) was co-expressed in *E. coli* cells with a fragment of *Hs.*INCENP (aa.835–904). The human Aurora B/INCENP complex, hereafter referred to as Aurora B for short, was purified and its kinase activity was confirmed towards the phosphorylation of histone H3 at Ser10 (Figure S1). Importantly, we found that Aurora B can hydrolyse ATP in the absence of peptide or protein substrates by luciferase-based luminescence assay (Figure 1(a)), suggesting that it also possesses an intrinsic ATPase activity. With this ATPase assay, we next carried out a small-molecule inhibitor screen with the bioactive compound library purchased from TopScience (Shanghai China). Among about 8,000 compounds, we identified a compound GSK650394 (Figure 1(b)) that selectively inhibits the ATPase activity of Aurora B, with an IC_50_ of 5.68 µM (Figure 1(c)), but only weakly inhibits another important mitotic kinase Haspin (IC_50_=41.4 µM, Figure S2). The results of *in vitro* kinase assay shows that this compound can also diminish the phosphorylation of histone H3 Ser10 by Aurora B (Figure 2(d)). Together, these results suggest that GSK650394 is a novel Aurora B inhibitor.

### 3.2. GSK650394 induces G2/M cell cycle arrest

GSK650394 was first discovered as an SGK inhibitor^28^ and has been shown to possess antiproliferative properties against many cancer cell lines including lung cancer^29^, thyroid cancer ^30^, and human prostate cancer^28^. However, the effects of this compound on HepG2 and Hela cells have not been determined. Moreover, Aurora B has also been shown to be overexpressed in hepatocellular^14^ and ovarian^15^ carcinomas. Aurora B inhibition by small-molecule inhibitors displayed anti-cancer activity in HepG2^25^ and Hela^40^ cells. We herein investigated the effect of GSK650394 in HeLa and HepG2 cells. We first tested if GSK650394 could exert any effect on the activity of endogenous Aurora B. To this end, we first monitored the changes of Aurora B activity across the cell cycle by detecting the phosphorylation of histone H3 Ser10. Western blot analysis of the samples obtained at a series of time points after thymidine release shows that the activity of Aurora B changes dynamically during the cell cycle, and reaches its maximum at 9 h after the G1/S phase (the metaphase) (Figure 2(a)). To evaluate the compound’s inhibitory effect, HeLa and HepG2 cells were synchronised at G1/S phase and then released into normal culture media for 9 h in the absence or presence of GSK650394. Compared to the control cells, cells treated with GSK650394 show significant attenuation of histone H3 phosphorylation. This effect is even more prominent for HepG2 cells, whose H3 phosphorylation is abolished by GSK650394 at 2.5 µM (Figure 2(b)). These results thus suggest that GSK650394 impairs the kinase activity of Aurora B in human cancer cells.

Since the kinase activity of Aurora B is required for normal cell cycle progression, we, therefore, asked whether GSK650394 would cause cell cycle arrest. HeLa and HepG2 cells synchronised at G1/S were released into normal media in the absence or presence of GSK650394 for 20 h, the resulting cells were collected and subjected to flow cytometric analysis. About 80% of cells progress normally through the mitosis and entered into the next G1/S phase in the absence of GSK650394 (Figure 2(c,d)). However, in the presence of GSK650394, we observed significant mitotic arrest in a concentration-dependent manner for both HeLa (Figure 2(c)) and HepG2 (Figure 2(d)) cells. In agreement with its ability of Aurora B inhibition for the two different cell lines, GSK650394 is more effective at inducing cell cycle arrest for HepG2 cells compared to HeLa cells.

### 3.3. GSK650394 attenuates cancer cells proliferation

**Figure 3.**
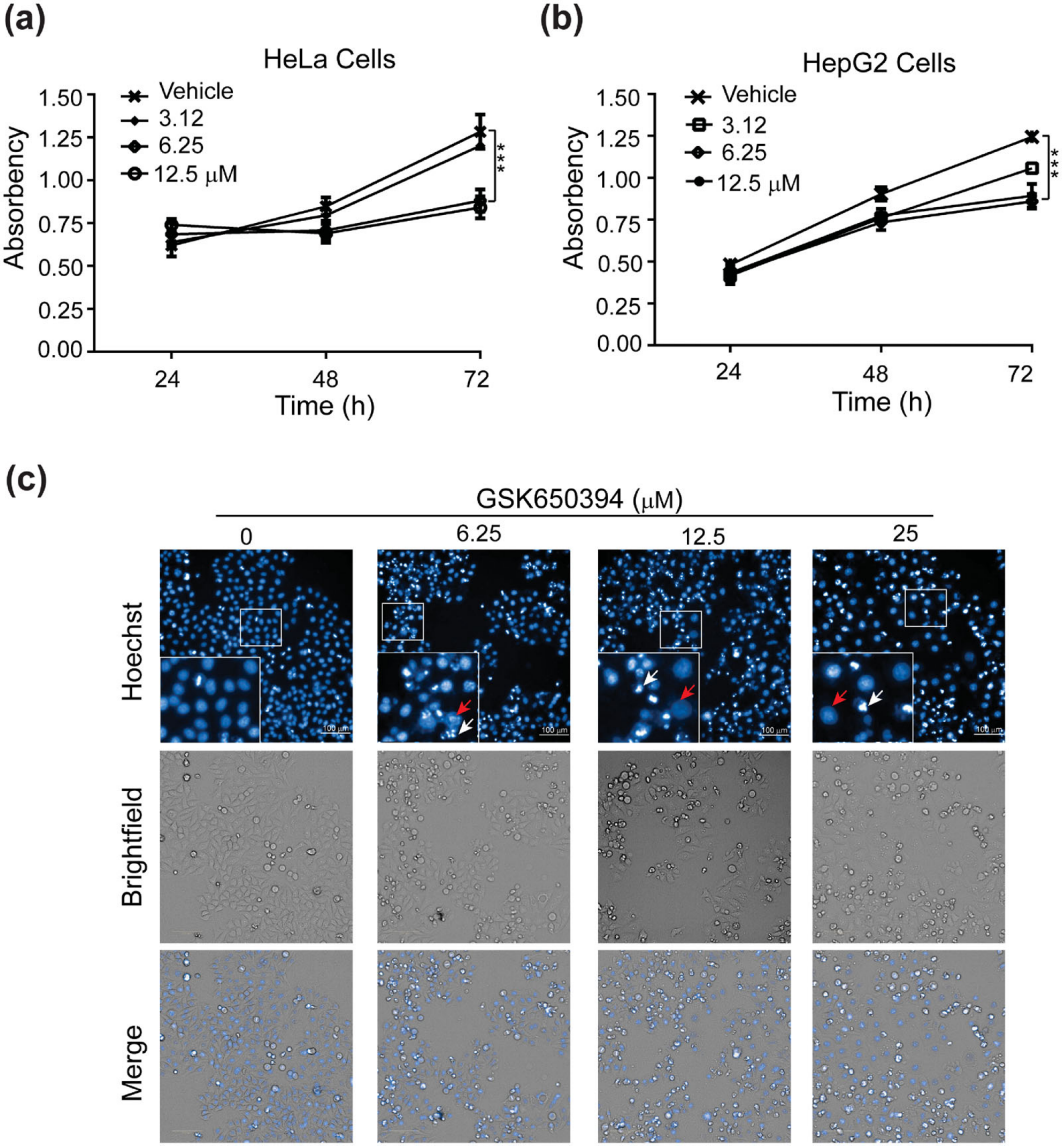
GSK650394 suppresses cancer cells proliferation. (a, b) MTT cell proliferation assay for HeLa (a) and HepG2 (b) cells. Cells were treated with indicated concentrations of GSK650394 for 24–72 h. After incubation with MTT solution, the number of viable cells was determined by measuring the absorbance at 490 nm. Values are means ± SD, *n* = 5. ***P < 0.001 vs. vehicle. (c) Effect of GSK650394 on the nuclear morphology of HeLa cells. Cells were treated with indicated concentrations of GSK650394 for 48 h and the nuclei were stained with DAPI. Outlined regions were magnified and shown in the left bottom corner. Red arrows indicated the expanded nuclei and white arrows for cell debris.

We next investigated the effect of GSK650394 on the proliferation of human cancer cells. HeLa and HepG2 cells were treated with various concentrations of GSK650394 for 24–72 h, and the number of viable cells was measured by MTT assay. Compared to the control cells, cells treated with GSK650394 show significant growth inhibition in a dose-dependent manner (Figure 3(a,b)). In addition, GSK650394 also inhibits the proliferation of LO2, MCF-7, and HELF cells with similar potencies (Table 1). A close examination of nuclear morphology reveals that the nuclei of drug-treated cells are either larger or smaller than the normal cells (Figure 3(c)), presumably resulting from gain or loss of chromosomes due to chromosome missegregation. In addition, we also observed a large quantity of cell debris upon the treatment of GSK650394 (Figure 3(c)), indicating cell death during drug treatment.

**Table 1.** Inhibition of GSK650394 against the proliferation of various cell lines.

Cell line	IC_50_ (µM)
HepG2	1.53 ± 1.88
LO2	1.75 ± 1.28
MCF-7	1.42 ± 1.19
HELF	6.14 ± 1.33
Hela	3.57 ± 1.38

### 3.4. GSK650394 inhibits the proliferation of *Aspergillus fumigatus*

**Figure 4.**
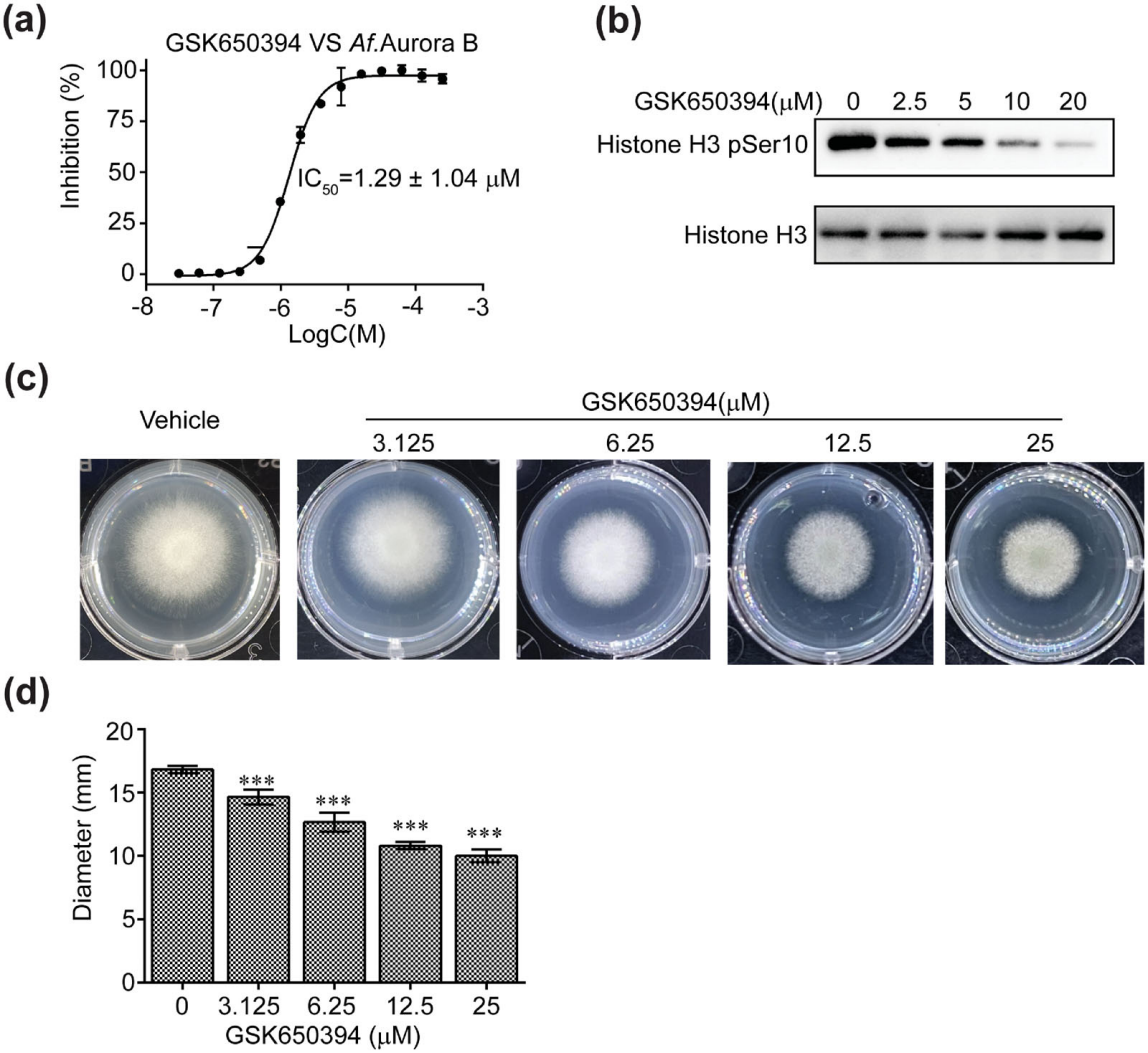
GSK650394 inhibits the growth of *Aspergillus fumigatus*. (a) Inhibition of GSK650394 on the ATPase activity of recombinant *Af.*Aurora B. The values were normalised to ATP-only control set as 100% inhibition. Values are means ± SD, *n* = 3. (b) Western blot analysis of histone H3 phosphorylation by the recombinant *Af.*Aurora B in the absence or presence of GSK650394. The immunoblots are representative of two independent experiments. (c) Effect of GSK650394 on the growth inhibition of *Aspergillus fumigatus* by using the plate assay. (d) Quantification of the diameters of *Aspergillus fumigatus* clonies in (c). Values are means ± SD, *n* = 3. ***P < 0.001 vs. vehicle.

Because the role of Aurora B in cell cycle regulation is conserved from fungus to humans, we next investigated the effect of GSK650394 on the proliferation of *Aspergillus fumigatus*. We first tested the inhibitory ability of GSK650394 on *Af.*Aurora B, which shares 51% sequence identity in the kinase domain with *Hs.* Aurora B. The recombinant kinase domain of *Af.* Aurora B was purified similarly to that of *Hs.*Aurora B. GSK650394 effectively inhibits both the ATPase (IC_50_ = 1.3 µM, Figure 4(a)) and the kinase activities of *Af.* Aurora B (Figure 4(b)). We then carried out a plate assay to assess the antifungal effect of GSK650394. To this end, a specified amount of *Aspergillus fumigatus* spore solutions were placed in the middle of the agar plate with or without GSK650394, and cultured at 37 °C. The growth rate of *Aspergillus fumigatus* was evaluated by the diameter of fungal clonies. Without treatment, the *Aspergillus fumigatus* cells grow fast and nearly spread over the whole plate within 30 h. However, when treated with GSK650394, the growth rate of *Aspergillus fumigatus* are markedly inhibited in a dose-dependent manner (Figure 4(c,d)), suggesting that GSK650394 has a potent anti-*Aspergillus fumigatus* efficacy.

### 3.5. GSK650394 is an ATP-competitive inhibitor

**Figure 5.**
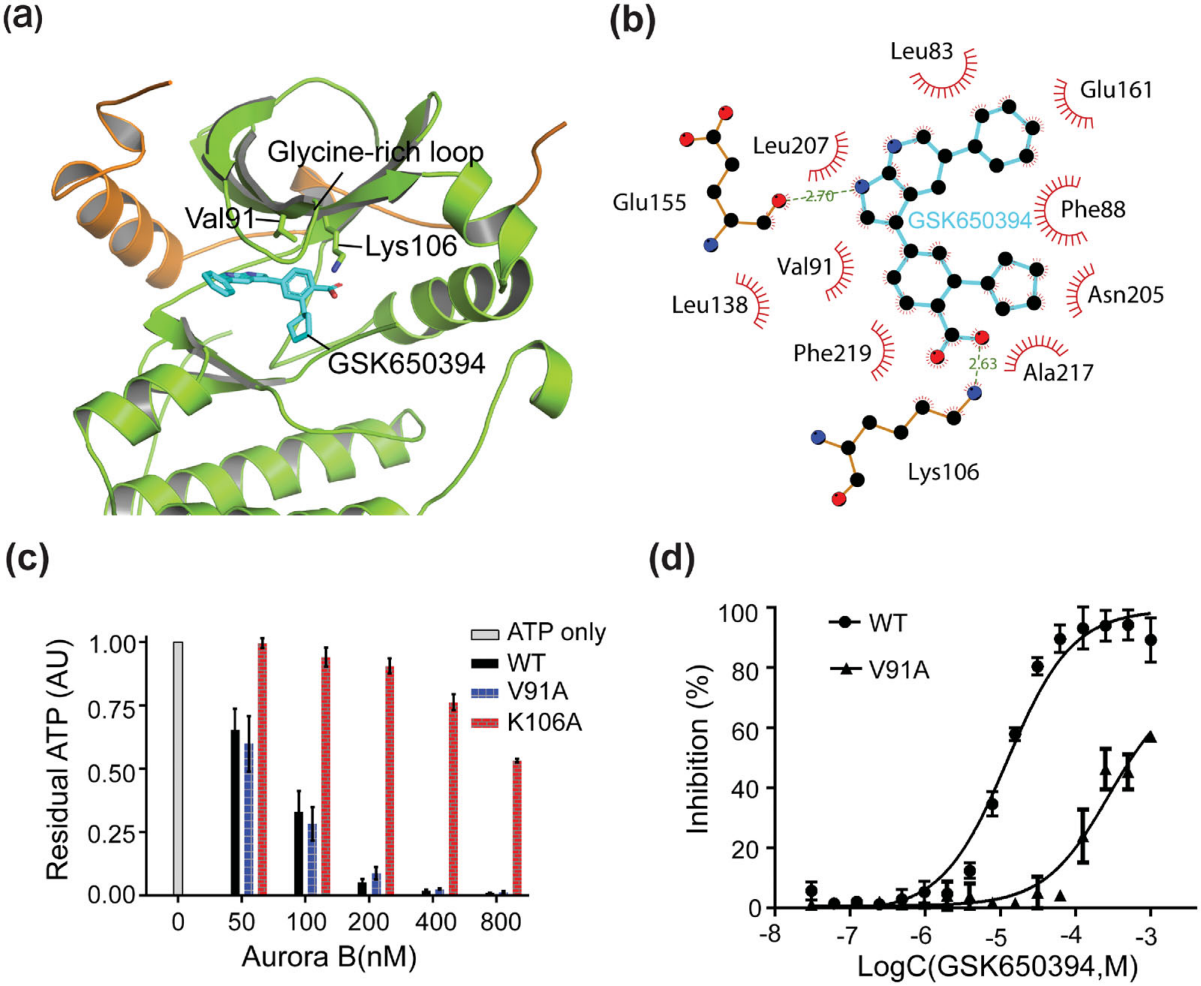
Molecular docking of GSK650394 to human Aurora B. (a) The best-calculated model of human Aurora B bound to GSK650394, with the crystal structure of human Aurora B/INCENP complex (PDB: 4AF3) as starting model. Aurora B was shown in a green cartoon, INCENP was in the orange cartoon, GSK650394 and its key interacting residues were shown in cyan sticks. (b) Detailed interaction between GSK650394 and Aurora B. The interaction was calculated using the program of LIGPLOT, with green dashed lines for hydrogen bonds, and spiked residues for hydrophobic contacts. GSK650394 was shown in the cyan stick. (c) Comparison of ATPase activity between wild type (WT) and mutant Aurora B proteins as measured by luciferase-coupled ATP assay. The luminescence of the ATP-only control was normalised to 1.0. (d) Effect of GSK650394 on the inhibition of wild-type (WT) and V91A Aurora B. The values were normalised to ATP-only control set as 100% inhibition. Values are means ± SD, *n* = 3.

We finally explored the molecular mechanism of GSK650394 in Aurora B inhibition. Despite repeated efforts, we were unable to obtain X-ray diffraction quality crystals of Aurora B/GSK650394 complex. To gain insight into their interacting mode, we thus conducted a molecular docking calculation using the published crystal structure of the human Aurora B/INCENP complex as starting model (PDB: 4AF3)^36^. To validate the docking study, we first performed a docking calculation of the published co-crystal structure of human Aurora B with a small molecule inhibitor VX-680^36^. As expected, the binding of VX-680 with Aurora B in the crystal structure can be reproduced in our docking calculation (Figure S3(A)), suggesting that our docking study is effective. We next carried out the docking of GSK650394 with human Aurora B. The model with the lowest binding energy was defined as the best docking result and was selected for further ligand binding analysis (Figure S3(B)). Comparison of the current best docking model and the crystal structure of Aurora B/INCENP/AMP-PNP complex (PDB: 4C2W) reveals that GSK650394 bind to the canonical nucleotide-binding site of Aurora B (Figure 5(a)). In the calculated model, the carboxyl group of GSK650394 forms a hydrogen bond with Aurora B Lys106, colliding with the binding of ATP γ − phosphate (Figure 5(b)). Mutation of Lys106 abolishes the catalytic activity of Aurora B (Figure 5(c)), suggesting that this residue is critical for the binding of both ATP and the inhibitor. In addition, the two nitrogen atoms on the azaindole group of GSK650394 forms a hydrogen bond with the main-chain carbonyl oxygen of Glu155, and the azaindole group, as well as the two aromatic rings, are also involved in extensive hydrophobic interactions with the side-chains of Leu83, Phe88, Val91, Leu138, Asn205, and Leu207. Mutation of Val91 close to the glycine-rich loop greatly diminishes the inhibitory potency of GSK650394 (IC_50_ >100 µM) without affecting the activity of Aurora B (Figure 5(c,d)). Thus, Val91 is mainly involved in the binding with GSK650394 rather than ATP. The structure of *Af.*Aurora B has not been determined yet, we thus built a structure model by SWISS-MODEL for this species. The modelled structure of *Af.*Aurora B is virtually identical to that of human Aurora B, with a root-mean-square (RMS) deviation of 1.2 Å (Figure S4(A)). Furthermore, the critical residues in ligand-binding pocket V91, Lys106, Glu155, Leu207 are conserved between the two species (Figure S4(B)). These observations are thus in good agreement with the above results that GSK650394 inhibited *A. fumigatus* and human Aurora B with similar potencies.

## 4. Discussion

Invasive fungal infections including candidiasis and aspergillosis are associated with considerable morbidity and mortality in immunocompromised individuals, such as cancer patients who are undergoing chemotherapy or radiotherapy. It is suggested that primary antifungal prophylaxis should be used in high-risk patients. Currently, Triazole antifungals such as itraconazole, voriconazole, and posaconazole are recommended as first-line drugs for aspergillosis treatment and prophylaxis, respectively^3^. However, in recent years, the emergence of azole-resistant *A. fumigatus* has been reported worldwide which can cause serious clinical problem^41^. Therefore, there is an urgent need to develop new classes of antifungal drugs. In this work, we identified a small-molecule inhibitor GSK650394 targeting the mitotic kinase Aurora B with anticancer and anti-*Aspergillus fumigatus* dual efficacies. Since many pharmaceutical companies are reluctant to develop new antifungal therapeutics due to limited financial returns, our work is expected to attract more interest from researchers and pharmaceutical companies in the development of anti-invasive fungal drugs and might provide new insights into the combinatory treatments of cancer and fungal infection.

As an inhibitor of serum- and glucocorticoid-regulated kinases (SGK)^28^, GSK650394 has been shown to inhibit the proliferation of various human cancer cells, such as lung cancer^29^, thyroid cancer^30^, and human prostate cancer^28^. Herein, we found that GSK650394 is a novel Aurora B inhibitor and has potent anti-cancer activity on HepG2 and Hela cells. These finding thus extends the application potential of this compound in cancer treatments. Furthermore, a previous study showed that SGK1 inhibition by GSK650394 could induce G2/M arrest, apoptosis, and autophagy in human prostate cancer^42^. Our results demonstrate that GSK650394 can diminish histone H3 S10 phosphorylation in HeLa and HepG2 cells, and induce significant G2/M cell cycle arrest. Given the critical role of Aurora B in cell cycle regulation, it is likely that the previously reported effect of GSK650394 on G2/M-phase cell cycle arrest could be, at least in part, related to the off-target inhibition of Aurora B.

By molecular docking and mutagenesis analyses, we demonstrate that GSK650394 is bound to the canonical nucleotide-binding site of Aurora B, with its carboxyl group competing ATP γ − phosphate for binding to Aurora B Lys106 and the large aromatic group occupies the hydrophobic pocket. Comparisons of the docking model with the crystal structures of Aurora B bound to AMP-PNP or the known Aurora kinase inhibitor VX-680 show that ATP interacts with Aurora B mainly through the hydrophobic residues in the deep pocket, including Leu83, Leu138, Tyr156 and Leu207. In addition to these contacts, GSK650394 also closely contacts residues Phe88 and Val91 outside the pocket. Mutation of Val91 near the glycine-rich loop does not affect Aurora B activity, but greatly diminishes the inhibitory effect of GSK650394 with >20-fold reduction. Importantly, residues Phe88 and Val91 have also been shown to be critical for the binding of VX-680^36^. Thus, residues Phe88 and Val91 could be considered as a binding hotspot for the design of selective inhibitors in the future.

It is well known that ATP is the primary carrier of energy in cells. Upon hydrolysis, it releases energy from the chemical bonds to fuel cellular processes. For example, ATP hydrolysis by motor proteins or DNA helicases can induce conformational changes and thus drive the translocation of these proteins. In addition, the protein kinases regulate various biological processes by transferring a phosphate group from ATP to amino acid residues like serine, threonine, or tyrosine. Interestingly, the mitotic kinases Aurora B, Haspin, and Bub1^32^ also possess intrinsic ATPase activity, producing free inorganic phosphate. It is currently unknown whether this energy-consuming activity has a physiological role in cells, further studies are needed to address this potentially interesting question.

## 5. Conclusions and perspectives

In summary, this study identified GSK650394 as a novel Aurora B inhibitor that could effectively suppress the proliferation of human cancer cells and attenuate the growth of the human pathogenic fungus *Aspergillus fumigatus*. The results showing here are expected to provide new insight into the combinational therapy of cancer and *Aspergillus fumigatus* infection. Future work may aim to test if the compound could also have effects on *Candida albicans*, the other important opportunistic pathogenic yeast. Moreover, because the carboxylate form of GSK650394 may be poorly absorbed by the gastrointestinal tract due to high polarity and negative charge of the carboxyl group, an ethyl ester modification might increase the bioavailability and *in vivo* efficacies of this compound.

## Supplementary Material

Supplemental Material
