# Supplementary material for: Discovery of a novel Aurora B inhibitor GSK650394 with potent anticancer and anti-aspergillus fumigatus dual efficacies in vitro
Source: J Enzyme Inhib Med Chem. 2021 Dec 11;37(1):109–17. doi: 10.1080/14756366.2021.1975693 (PMC8667888; doi:10.1080/14756366.2021.1975693)
Supplement: Supplemental Material [file IENZ_A_1975693_SM2444.pdf]

Supplementary Figures

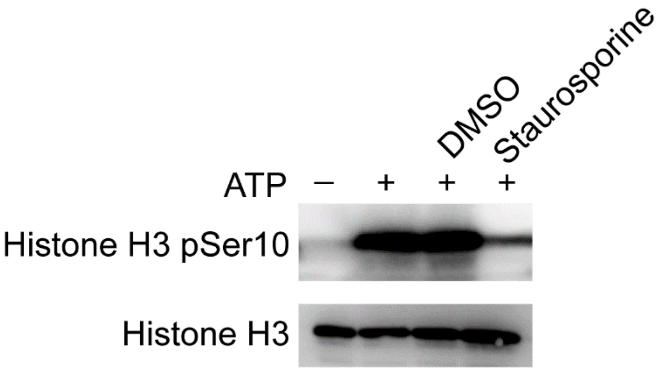

Fig. S1 Phosphorylation of histone H3 by the recombinant human Aurora B.

200 nM of Aurora B protein was pre-incubated with reaction buffer, DMSO or 10 μM staurosporine, and then 6 μg of bulk histones, with or without 100 μM ATP, were added to initiate reaction. The reaction mixtures were separated on SDS-PAGE followed by immunoblot analysis with indicated antibodies.

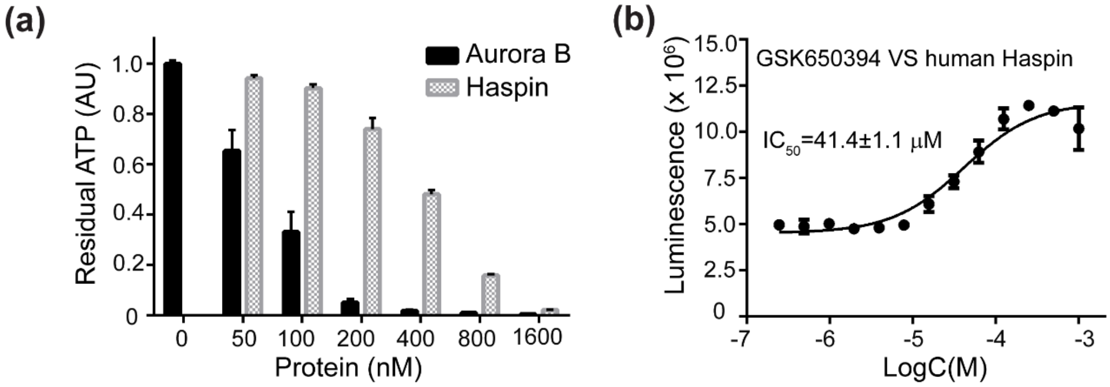

Fig. S2 Effect of GSK650394 on human Haspin. (a) Comparison of ATPase activity between Aurora B and Haspin. The luminescence of ATP only control was normalized to 1.0. The values were normalized to ATP-only control set as 100% inhibition. Values are means ± S.E., n = 3. (b) Inhibition of human Haspin ATPase activity by GSK650394.

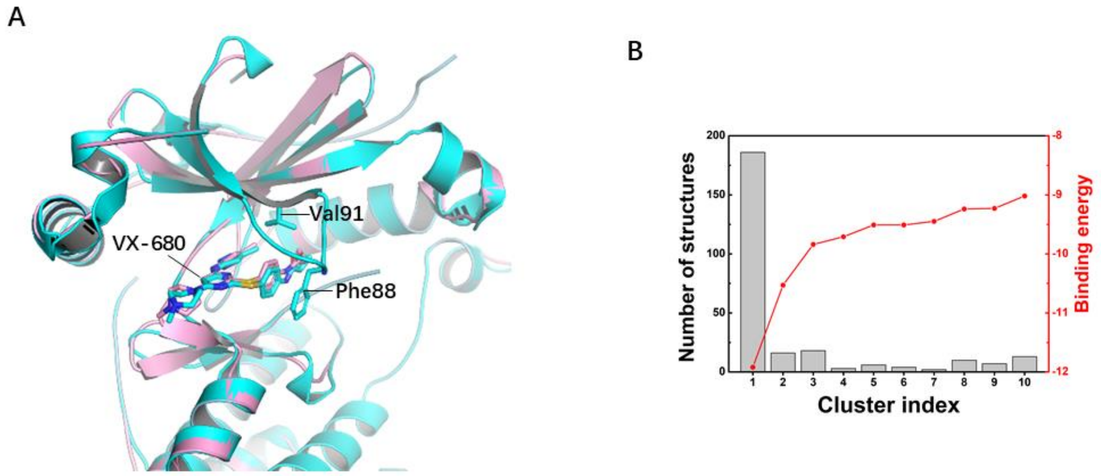

Fig. S3. Molecular docking studies of human Aurora B with small molecule inhibitors. A. Superposition of the calculated model (pink) and co-crystallized structure (cyan) of human Aurora B with the reported inhibitor VX-680; **B.** Cluster index of the docking result of human Aurora B with GSK650394. The numbers of structure and mean binding energy (in kcal/mol) for each cluster are represented by grey column and red line, respectively. Cluster No.1 had the lowest binding energy and was selected for ligand binding analysis. All the docked structures were clustered using the default parameters implemented in AutoDockTools.

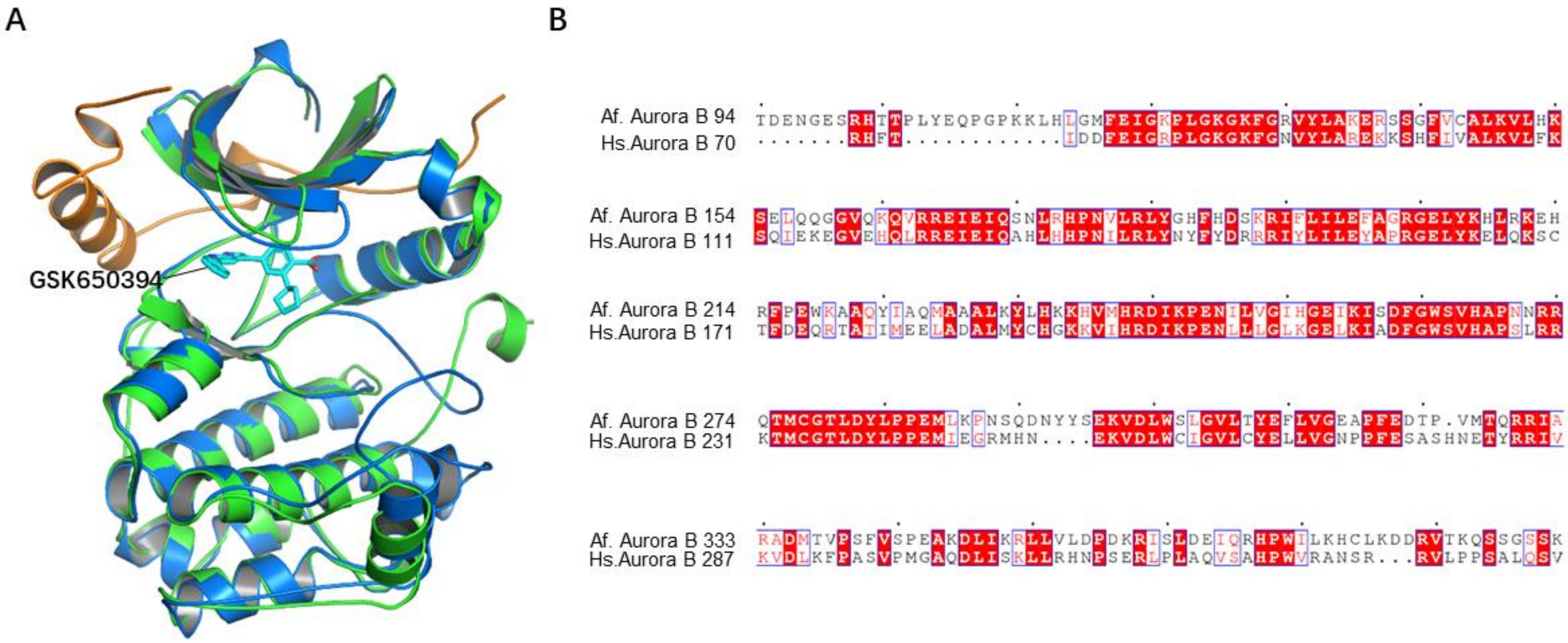

Fig.S4. Comparison of human and *A.fumigatus* Aurora B proteins. A. Comparison of crystal structure of *Hs.*Aurora B (green) and the structure model of *Af.*Aurora B (blue) generated by SWISS-MODEL (https://swissmodel.expasy.org); **B.** Sequence alignment between human and *A.fumigatus* Aurora B proteins. The alignment is generated with the online server of ESPript 3.0.
